# Polyphasic Taxonomic Analysis Establishes *Mycobacterium indicus pranii* as a Distinct Species

**DOI:** 10.1371/journal.pone.0006263

**Published:** 2009-07-16

**Authors:** Vikram Saini, Saurabh Raghuvanshi, Gursaran P. Talwar, Niyaz Ahmed, Jitendra P. Khurana, Seyed E. Hasnain, Akhilesh K. Tyagi, Anil K. Tyagi

**Affiliations:** 1 Department of Biochemistry, University of Delhi South Campus, New Delhi, India; 2 Interdisciplinary Centre for Plant Genomics and Department of Plant Molecular Biology, University of Delhi South Campus, New Delhi, India; 3 Talwar Research Foundation, New Delhi, India; 4 Pathogen Biology Laboratory, School of Life Sciences, University of Hyderabad, Hyderabad, India; 5 Institute of Life Sciences, University of Hyderabad, Hyderabad, India; 6 Jawaharlal Nehru Centre for Advanced Scientific Research, Jakkur, Bangalore, India; 7 National Institute of Plant Genome Research, Aruna Asaf Ali Marg, New Delhi, India; Cairo University, Egypt

## Abstract

**Background:**

*Mycobacterium indicus pranii* (*MIP*), popularly known as *Mw*, is a cultivable, non-pathogenic organism, which, based on its growth and metabolic properties, is classified in Runyon Group IV along with *M. fortuitum*, *M. smegmatis* and *M. vaccae*. The novelty of this bacterium was accredited to its immunological ability to undergo antigen driven blast transformation of leukocytes and delayed hypersensitivity skin test in leprosy patients, a disease endemic in the Indian sub-continent. Consequently, *MIP* has been extensively evaluated for its biochemical and immunological properties leading to its usage as an immunomodulator in leprosy and tuberculosis patients. However, owing to advances in sequencing and culture techniques, the citing of new strains with almost 100% similarity in the sequences of marker genes like 16S rRNA, has compromised the identity of *MIP* as a novel species. Hence, to define its precise taxonomic position, we have carried out polyphasic taxonomic studies on *MIP* that integrate its phenotypic, chemotaxonomic and molecular phylogenetic attributes.

**Methodology/Principal Findings:**

The comparative analysis of 16S rRNA sequence of *MIP* by using BLAST algorithm at NCBI (nr database) revealed a similarity of ≥99% with *M. intracellulare*, *M. arosiense*, *M. chimaera*, *M. seoulense*, *M. avium* subsp. *hominissuis*, *M. avium* subsp. *paratuberculosis* and *M. bohemicum*. Further analysis with other widely used markers like *rpoB* and *hsp65* could resolve the phylogenetic relationship between *MIP* and other closely related mycobacteria apart from *M. intracellulare* and *M. chimaera*, which shares ≥99% similarity with corresponding *MIP* orthologues. Molecular phylogenetic analysis, based on the concatenation of candidate orthologues of 16S rRNA, *hsp65* and *rpoB*, also substantiated its distinctiveness from all the related organisms used in the analysis excluding *M. intracellulare* and *M. chimaera* with which it exhibited a close proximity. This necessitated further analysis of *MIP* with more sensitive and segregating parameters to ascertain its precise taxonomic position as a new species. The analysis of *MIP* and its comparison with other mycobacterial reference strains based on cellular and biochemical features, growth characteristics and chemotaxonomic studies like FAME profiling confirmed that *MIP* is uniquely endowed with diverse metabolic attributes that effectively distinguishes it from all the closely related mycobacteria including *M. intracellulare* and *M. chimaera*.

**Conclusion:**

The results presented in this study coupled with the non-pathogenic nature and different biochemical and immunomodulatory properties of *MIP* affirm it as a distinct species belonging to *M. avium* complex (*MAC*). It is further proposed to use an earlier suggested name *Mycobacterium indicus pranii* for this newly established mycobacterial species. This study also exemplifies the growing need for a uniform, consensus based broader polyphasic frame work for the purpose of taxonomy and speciation, particularly in the genus *Mycobacterium*.

## Introduction

In the late seventies, a *Mycobacterium*, coded as *‘w’*, was selected by Prof. G. P. Talwar and his colleagues at the All India Institute of Medical Sciences, New Delhi, from a panel of known atypical mycobacteria for its ability to evoke cell mediated immune responses against *M. leprae* in multibacillary lepromatous leprosy patients, normally anergic to *M. leprae* 1. This *Mycobacterium ‘w’*, when used as an adjunct to the standard multidrug therapy against multibacillary leprosy patients, exhibited a significantly enhanced bacillary clearance thereby shortening the full recovery time of patients [2]–[4]. It has emerged as a powerful immunomodulator in one of the largest clinical trials in India involving approximately 30,000 household contacts of leprosy patients 5. ‘*Mw*’, commercially available as “Immuvac” vaccine, shares antigens with *M. leprae* and *M. tuberculosis* and provides protection against *M. tuberculosis* infection in both BCG responder (Balb/c, C57BL/6 NCrl and C3H/He NCrl) and non-responder (CBA/N) genetically distinct strains of mice [6], [7]. Moreover, a recent study in mice has confirmed its immunogenicity and protective efficacy against *M. tuberculosis* infection in both live as well as heat-killed form [8]. In the light of its distinctive immunomodultory actions and a plausible ambiguity of nomenclature with a recently emerged hyper virulent Beijing strain *Mycobacterium tuberculosis* ‘W’, it was suggested to use the name *Mycobacterium indicus pranii* (*MIP*) for this bacterium [9].

However, despite the emerging prominence of *MIP* as a broad-spectrum vaccine candidate, there have been limited attempts on its molecular characterization by genotypic analysis barring the study of a standard gene locus *hsp65* [10]. With the advent of new sequencing technologies and better culture techniques, there has been an increased awareness about the diversity within the microbial world, especially in genus *Mycobacterium*. Consequently, many new species have recently been notified that share nearly 100% similarity with the characteristic molecular signatures of *MIP* [11]–[13]. Since such an extreme sequence conservation at species level is well documented in the case of *Mycobacterium*, further analysis of *MIP* would be sagacious to have an explicit understanding of taxonomic identity and specific physiological attributes of this bacterium, particularly in the context of evolution and speciation. For this, extensive polyphasic taxonomic studies were undertaken pertaining to its phenotypic (size, type and morphology), chemotaxonomic (whole cell fatty acid analysis), molecular (presence or absence of genomic markers) and phylogenetic characterization based on concatenation of representative orthologues of *MIP* like 16S rRNA, *hsp65* and *rpoB*, which have been widely used for species differentiation studies in mycobacteria [14]. Here, we describe the results of these studies, evaluate these findings in the light of taxonomy and evolution of mycobacteria and define the precise taxonomic position of *MIP* as an independent species belonging to *M. avium* complex.

## Results

### Purity of culture, colony morphology and molecular identity

The growth of *MIP* on Middlebrook (MB) 7H11 agar as uniform colonies indicated the purity of the culture. The colonies were 1–2 mm in size, smooth, convex, monotypic, raised, shiny, round and nonpigmented. They were not arranged in any definite pattern. No cording was observed. Molecular identity of *MIP* was established by PCR amplification of *hsp65* gene with *MIP* genomic DNA as template. A ∼440 bp amplicon with *MIP* specific nucleotide substitutions at positions 94, 121, 130 and 286 bp authenticated the strain used in this study [10]. These substitutions rendered it distinct from *M. tuberculosis* H37Rv, *M. bovis*, *M. bovis* BCG, *M. leprae*, *M. avium*, *M. intracellulare, M. scrofulaceum, M. paratuberculosis, M. kansasii, M. gastri, M. gordonae, M. shimoidei, M. malmoense, M. haemophilum, M. nonchromogenicum, M. trivale, M. marinum, M. flavescens, M. simiae, M. sculgai, M. xenopi, M. asiaticum, M. aurum, M. smegmatis, M. vaccae, M. fortuitum, M. chelonae* and *M. abscessus* [10].

### Growth pattern and biochemical features

**Figure 1:**
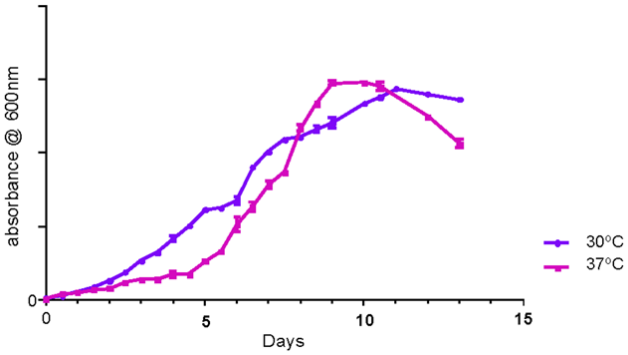
Growth rate analysis of *MIP*: *MIP* was cultured in MB7H9-ADC medium at 30°C and 37°C. The A_600nm_ of liquid culture of *MIP* was plotted against time to analyze the pattern of *MIP* growth. Growth was monitored by measuring the change in the value of A_600nm_ over time. Each experiment was performed with replicates and error bars for each time point are shown. A typical growth curve with three distinct phases was generated with culture becoming saturated in 8 to 10 days.

*MIP* showed no apparent growth on nutrient and MacConkey agar; however, the growth on Lowenstein Jensen (LJ) slant was observed in 4–6 days. On MB7H11 agar, *MIP* colonies appeared in between 6–8 days. It did not produce any pigment either in light or dark. Apparently, *MIP* grows relatively faster, when compared to *M. tuberculosis* (>3 weeks), *M. seoulense* (>3 weeks), *M. arosiense* (>3 weeks), *M. bohemicum* (4–6 weeks) and the members of *MAC* complex like *M. intracellulare*, *M. avium* subsp. *hominissuis*, *M. avium* subsp. *paratuberculosis* and *M. chimaera* (>2 weeks) [11]–[13], [15], [16]. However, when compared to usual fast growers, such as *M. smegmatis* (<2 days), *M. pheli* (<5 days) and *M. vaccae* (<5 days), *MIP* actually grows considerably slowly notwithstanding its several characteristics usually associated with rapid growers such as the ability to grow in 5% NaCl (Table 1) [17]. The growth curve analysis of *MIP* in MB7H9 broth revealed that it reached a saturation phase in 8 to 10 days (Figure 1). There was no apparent difference in growth and colony forming time of *MIP* at 30°C and 37°C on MB7H11 agar. However, in broth culture (MB7H9), it grew faster at 37°C and reached the saturation phase earlier in comparison to 30°C inspite of a relatively prolonged lag phase. *MIP* has been predicted to be a fast grower based on its growth on LJ medium, Dubos agar and in Sauton's medium [18], [19]. Thus, *MIP* seems to share properties which are exclusive to either slow growers or fast growers reflecting upon its unique position, wherein it grows faster than the typical slow growers belonging to *MAC* and slower in comparison to classical fast growers belonging to Runyon Group IV like *M. smegmatis*. Thus, *MIP* could be differentiated from the members of *MAC* by virtue of its faster growth rate and colony forming time on MB7H11 agar.

**Table 1:** Comparative analysis of biochemical characteristics of *MIP* with other related mycobacteria.

Sr. No.	Tests*	*M. chimaera*	*M. seoulense*	*M. arosiense*	*M. intracellulare*	*MIP*	*M. vaccae*	*M. fortuitum*	*M. smegmatis*	*M. pheli*
1	Rate of growth	S	S	S	S	R	R	R	R	R
2	Pigment	S	S	S	S	N	N	N	N	S
3	Temperature									
	a) 25°C	+	+	−	+	+	+	+	+	+
	b) 37°C	+	+	+	+	+	+	+	+	+
	c) 45°C	−	−	+	−	+	−	−	+	+
4	Colony type	smooth	smooth	smooth	variable	smooth	smooth	smooth	smooth	rough
5	Growth on MacConkey agar	−	−	−	−	−	−	+	−	−
6	Growth on 5% NaCl	−	−	−	−	+	+	+	+	+
7	Tween 80 hydrolysis (5 days)	−	−	−	−	−	+	−	+	+
9	Urease production	−	−	−	−	−	+	+	+	+
10	Nitrate reduction	−	+	+	−	+	+/−	+	+	+
12	Aryl sulfatase (3 days)	−	−	−	−	−	−	+	−	−
13	Aryl sulfatase (14 days)	-na-	−	−	−	+	+	+	+	+
14	Catalase semi-quantitative	−	−	−	−	+	+	+	+	+
15	Heat resistant Catalase	+	−	+	+/−	+	+	+	+	+

(R): rapid growers, (N): no pigmentation, (S): scotochromogene, (+): positive, (−): negative, (+/−): presence in some strains of species but not in all, (na): data not available, variable: inconsistent colony type (smooth/rough/translucent/opaque) [* as per the tests performed in Saxena et al. [18], Katoch VM [19] and this study].

Biochemically, *MIP* was negative for niacin test, Tween 80 hydrolysis and urease production and positive for semi quantitative catalase and heat resistant catalase, tellurite reduction and for sodium salicylate degradation [18], [19]. *MIP* could grow at 25°C and 45°C and was found to be resistant to isoniazid (10 µg/ml). Besides, the bacillus could reduce nitrates to nitrites and could also utilize sodium nitrate and sodium nitrite as nitrogen sources. The organism did not grow on fructose and arabinose as the only source of carbon. The detailed biochemical properties of *MIP* and their comparative analysis with related mycobacteria are depicted in Table 1.

### Molecular and phylogenetic analysis of *MIP* reveals its proximity with opportunistic mycobacteria of *M. avium* complex

**Figure 2:**
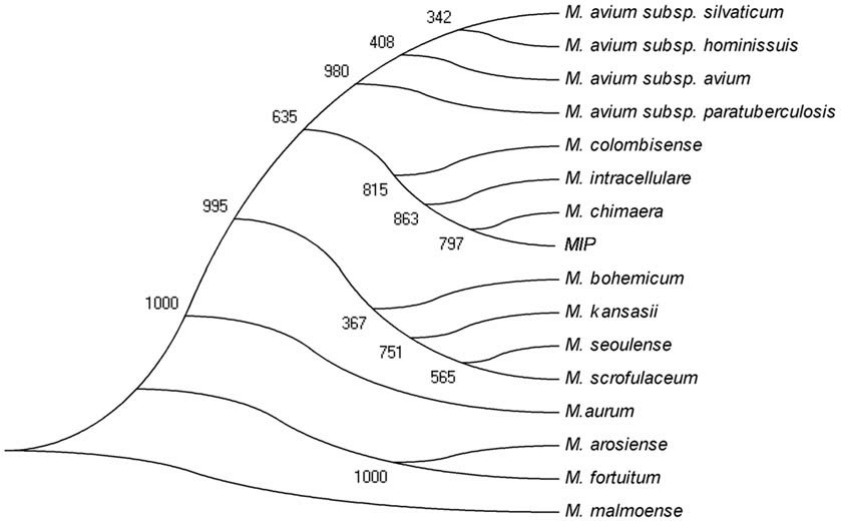
Phylogenetic positioning of *MIP*: The Phylogenetic positioning of *MIP* with respect to other members of genus *Mycobacterium* was performed by making concatenated tree of 16S rRNA, *rpoB* and *hsp65* involving members of *MAC* and other environmental isolates, which are close to *MIP*. The alignment was carried out using clustal x ver 1.81[50] and a phylogenetic tree rooted to *M. malmoense* was constructed using Neighbor joining (NJ) method with 1,000 bootstrap iterations [51].

The BLAST based similarity searches of 16S rRNA of *MIP* with nr (non-redundant) database at NCBI (http://blast.ncbi.nlm.nih.gov/Blast.cgi), revealed a similarity of ≥99% displaying very limited mismatches with *M. intracellulare* (0.07%), *M. arosiense* (0.34%), *M. seoulense* (0.68%), *M. avium* subsp. *hominissuis* (0.75%), *M. avium* subsp. *paratuberculosis* (0.75%), *M. chimaera* (0.9%) and *M. bohemicum* (0.9%). All of these mycobacteria belong to *MAC* group of organisms except *M. bohemicum* and *M. seoulense*, which are closer to *M. scrofulaceum* and *M. kansassi* [11]. To further discriminate *MIP* from rest of the species, two widely used molecular chronometers namely, *rpoB* and *hsp65* were also evaluated [20], [21]. It has been reported that a sequence similarity of complete *rpoB* gene <97.7% correlates with an ANI (average nucleotide identity between two organisms) value of <94.3% and DDH (DNA: DNA Hybridization) value of <70%, which are the taxonomic benchmarks to assign species status with respect to intraspecies comparisons [22]. The comparison of *MIP rpoB* gene with corresponding orthologues from completed mycobacterial genome sequences revealed a percentage nucleotide similarity of 96% with *M. avium* subsp. *hominissuis*, 95% with *M. avium* subsp. *paratuberculosis* followed by 91% with *M. marinum*, thereby establishing the distinctiveness of *MIP* from *M. avium* subsp. *paratuberculosis* and *M. avium* subsp. *hominissuis* (Supplementary Figure S1). With *M. intracellulare*, *M. chimaera*, *M. bohemicum*, *M. arosiense and M. seoulense*, the percentage similarity of *MIP* was found to be 99.1%, 99.3%, 93%, 95% and 92%, respectively on comparison of their partial *rpoB* sequences, while a similarity of 99.7%, 98.8%, 93%, 95% and 95%, respectively was observed on comparing with corresponding *hsp65* sequences. The phylogenetic analysis involving *MIP*, all known members of *M. avium* complex along with environmental mycobacteria using concatenated loci of 16S rRNA, *hsp65* and *rpoB* genes as genotypic markers effectively showed that *MIP* was distinct from all other members of *MAC* except *M. intracellulare* and *M. chimaera* with which it showed an apparent proximity (Figure 2). These observations substantiated the importance of these genotypic markers in phylogenetic studies; nonetheless, the very few substitutions exhibited by these marker genes pointed out the need for more segregating parameters to further delineate the heterogeneity in *MAC*.

### Chemotaxonomic investigations reveal *MIP* to be different from all known members of *MAC* including *M. intracellulare* and *M. chimaera*

Chemotaxonomic investigations by FAME analysis have often played a cardinal role in resolving inadvertencies in case of taxonomic investigations in bacteria including the ones belonging to *Mycobacterium* as it examines the features at whole organism level [23], [24]. FAME (fatty acid methyl ester) analysis is a very sensitive approach, which proficiently reflects on biochemical and physiological attributes of an organism to correctly define its precise taxonomic position [25]–[27]. Hence, we analyzed *MIP* for the presence of FAME and compared it with the fatty acid profiles of other mycobacteria (Table 2). This comparative analysis demonstrated the predominant fatty acids of *MIP* as summed feature III that corresponds to 20:0 alcohol/19:0 cycloprop *ω*10c and/or 19:0 cycloprop *ω*8c, comprising 67.25% of total fatty acids content analyzed. Summed feature II comprised of 13.89% of total fatty acids content analyzed and corresponds to 17:1 *ω*7c/18: 0 alcohol/17:1*ω*6c/17: cyclopropane. Similarly, summed feature I was represented by <1% of total fatty acids content analyzed and comprised of 8-Me-16:0/10-Me-16:0 as given in Table 2. Thus, *MIP* could be distinguished from *M. intracellulare* not only by the presence of a higher fraction of fatty acid content as summed features but also by the presence of 18:1 *ω*7c (1.37%) and the absence of 20:0 fatty acids (Table 2). Similarly, *M. chimaera* could be typified by the relative preponderance of 18:1 *ω*9c (18.68%) and presence of 16:1 *ω*10c (5.29%), which is absent in *MIP* [17]. The comparative FAME analysis, thus, confirmed that *MIP* harbors unique metabolic machinery, which differentiates it significantly from all other mycobacteria used in this study including *M. tuberculosis*, *M. avium*, *M. intracelluare*, *M. chimaera*, *M. arosiense*, *M. seoulense* on the basis of biochemical parameters. Further analysis of *MIP* based on the measurement of evolutionary distance by using the FAME mycobacterial library (MIDI Sherlock, USA, which matches the sample's composition with the stored FAME patterns of various mycobacterial species to provide a relative distance from the “mean” of fatty acid population of the sample) showed *MIP* to be nearest to *M. scrofulaceum* (Distance = 21.095), followed by *M. aurum* (Distance = 32.03) and *M. fortuitum* (Distance = 84.4). It is noteworthy here that *M. aurum* and *M. fortuitum* are fast growers while *M. scrofulaceum* is classified as a slow growing organism.

**Table 2:** Comparative analysis of *MIP* with other related mycobacteria by FAME.

Sr. No.	Feature Name	*M. bohemicum*	*M chimaera*	*MIP*	*M. intracellulare*	*M. scrofulaceum*	*M. aurum*	*M. fortuitum*	*M. tuberculosis*	*M. avium*
1	12:0	-	0.24	0.12	-	-	-	-	-	-
2	14:0	2.55	7.43	3.5	4.69	4.44	5.93	7.1	1.33	3.6
3	15:0	0.53	0.49	0.8	0.58	0.46	-	0.55	0.53	0.6
4	16:0	26.93	24.10	4.68	35.32	36.32	30.12	43.54	39.21	34.98
5	17:0	0.52	0.30	-	-	0.5	-	0.52	2.45	-
6	18:0	3.46	2.27	0.21	4.48	7.19	2.66	4.43	10.6	3.98
7	10Me-18:0 TBSA	7.48	8.26	1.75	13.28	6.3	9.09	14.35	19.79	13.31
8	20:0	0.67	0.52	-	0.6	0.85	2.23	0.63	1.16	0.47
9	16:1 *ω*6c	-	-	3.04	7.23	6.06	5.58	7.46	3.41	6.43
10	16:1 *ω*7c	2.15	1.62	0.45	1.64	1.56	-	0.52	-	1.38
11	16:1 *ω*9c	1.60	1.54	0.27	0.71	0.57	2.19	0.65	-	1.08
12	18:1 *ω*7c	-	-	1.37	-	-	-	-	-	-
13	18:1 *ω*9c	24.42	18.68	2.44	17.52	21.1	27.35	19.25	19.71	19.38
14	*Summed Feature I	-	-	0.24	0.78	-	-	0.62	0.53	0.64
15	*Summed Feature II	-	-	13.89	3.01	5.76	10.01	-	-	1.75
16	*Summed Feature III	-	-	67.25	9.98	8.56	3.74	-	-	12

The FAME profile for *MIP* was generated by using Gas Liquid Chromatography and compared with the profiles of other mycobacteria in the FAME database of Microbial Identification System (MIDI, Inc., Newark, Del.).Values represent percentage amount of total fatty acids. [*Summed features consist of one or more fatty acids that could not be separated by the Microbial Identification System. Summed feature III: 20:0 alcohol/19:0cycloprop*ω*10cand/or 19:0 cycloprop *ω*8c; Summed feature II: 17:1 *ω*7c/18:0 alcohol/17:1*ω*6c/17: cyclopropane; and summed feature I: 8-Me-16:0/10-Me-16:0; TBSA- Tuberculostearic acid].

## Discussion

The classification of organisms into species with shared traits and niche preferences constitutes the cornerstone of the microbial world and is fundamental to efficiently organize and disseminate information about microbial diversity. The determination of molecular sequences and the understanding that they could be employed to differentiate organisms have revolutionized the perception of microbial diversity. The advent of new sequencing and culture methods has led to the identification of many new strains and availability of sequencing data of their marker genes. While this has made identification of new species easier in some cases, the consideration of arbitrary cutoff values based on similarity in selected genes like 16S rRNA (presently 99% with16S rRNA) as a yardstick to confer species status may sometimes be fraught with the danger of losing out on microbial diversity. A phylogenetic tree constituted from a set of genes essentially infers evolutionary histories of these genes, which may not necessarily reflect on the descent of species [28]. This observation is especially more relevant in case of organisms belonging to genus *Mycobacterium* which are clonal in nature and have very restricted nucleotide substitution rates [29].

With comparison of 16S rRNA gene, an extremely powerful tool and by far the single most common molecular technique presently used for bacterial species identification, *MIP* shows greater than 99% similarity with *M. intracellulare*, *M. arosiense*, *M. seoulense*, *M. chimaera, M. avium*, subsp. *hominissuis*, *M. avium* subsp. *paratuberculosis* and *M. bohemicum*. However, the maximum proximity was apparent with *M. intracellulare and M. chimaera* and was marked by extreme conservation (>99%) even on comparison of other genotypic markers, such as *rpoB* and *hsp65*. This gives the impression that *MIP* is related to these strains or is a derivative or sequevar of *M. intracellulare*, which appears to be inaccurate in the backdrop of the scientific evidence presented in this study. *M. intracellulare* besides being a known pathogen and a slow grower also does not grow in 5% NaCl. Unlike *MIP*, *M. intracellulare* does not reduce nitrate, a trait that it shares with *M. avium*, *M. chimaera* and *M. scrofulaceum* [12]. Similarly, *M. arosiense*, despite having extremely limited divergence with *MIP* on comparison of its marker genes used in this study, is a pathogenic scotochromogen that grows optimally at 42°C and takes more than 14 days for visible colonies to appear on 7H11 agar, that too with a heavy inoculum [12]. Thus, it is quite perspicuous that *MIP* harbors different biochemical traits from the organisms, which appear to be its close relatives on the basis of phylogenetic analysis based on marker genes (Table 1).

However, since based on the phylogenetic markers, the similarity ascertained between different orthologues was close to 100%, it was also evident that more sensitive and differentiating parameters would be required, if we have to further ascertain the significance of these small differences observed in the phylogenetic comparisons. Hence, as a next step, the FAME analysis of *MIP* was carried out. It offered the obvious advantages that: i) FAME analysis reflects on the biochemical and physiological attributes of the associated organisms rather than on the mutations in the genes encoding the candidate orthologues in order to correctly define their precise taxonomic position; and ii) it offers a highly reproducible value based on the comparisons with other members of the genus [23], [24]. Its significance became apparent, when the FAME profile of *MIP* was compared with its counterparts from several other members of family *Mycobacteriaceae*. The FAME pattern for *MIP* is different from *M. intracellulare*, *M. arosiense* and *M. chimaera*, the organisms closest to *MIP* on the basis of comparison of 16S rRNA sequence (Table 2). Incidentally, *M. intracellulare* and *M. arosiense* share a similar FAME profile marked by predominance of 16:0, 10-methyl 18:0 TBSA (tuberculostearic acid) and 18:1 *ω*9c as major fatty acids and can not be differentiated exclusively on the basis of FAME analysis [12]. However, it became clear that *MIP* possessed a FAME profile that was visibly distinct from the rest of the organisms available in FAME database although it showed certain proximity with *M. scrofulaceum* followed by fast growing *M. aurum*, *M. fortuitum* and other rapid growers (Table 2). From these observations, it was obvious that *MIP* possesses unique pathways of fatty acid synthesis probably reflecting on its need for a saprophytic life style. It is noteworthy here that lipids and fatty acids are known to have immunomodulatory activity [30], [31]. Besides, they are also involved in stimulation of cytokine production, proliferation of human T lymphocytes and in the activation of protein kinases [32]. It is tempting to speculate the role of these novel fatty acids in the immunomodulatory activity of *MIP* although this merits specific immunological investigations.

An important insight emerging from the above discussion is that reliance on a single identification system, whether phenotypic, genotypic or chemotaxonomic, may not be appropriate and can undervalue the microbial diversity thereby defying the overall rationale of taxonomy. This point bears special relevance in the case of genotypic taxonomy, which is based on the application of conserved housekeeping genes as markers. The usage of candidate marker genes in taxonomy is underpinned with a notion that these genes may correctly represent the entire genomic complexity of the species and hence can be good surrogate to define the species. However, it is being increasingly realized that this notion may not be absolutely correct [33], [34]. This point is specifically more pertinent in the case of mycobacteria, which are organisms of high biomedical prominence that share a similarity up to 99.95% even at the comparison of their whole genome sequences [35]. The literature is replete with the reports of mycobacteria which have been assigned the species status despite sharing almost 100% similarity in their marker genes [33], [36]. 16S rRNA gene, which has been preeminent in the advancement of bacterial taxonomy and has been the most widely used marker, reveals an identity of 100% (*M. kansasii* and *M. gastri*), 99.9% (*M. malomense* and *M. szulgai*) and 99.9% (*M. microti* and *M. bovis*), on its comparative analysis between mycobacterial species. The analysis of two other extremely popular genotypic markers *hsp65* and *rpoB* revealed a similarity of 99.5% and 99.6%, respectively between *M. marinum and M. ulcerans* and 99.5% and 99.9%, respectively between *M. intracellulare* and *M. chimaera*. *M. chimaera* in fact shows 100% similarity with *M. intracellulare* serovars type 7, although it is a distinct species. Thus, the resolution of these markers has been further compromised because of the heterogeneity in *M. avium* complex. Nonetheless, the species status has been accorded in these cases based on the mounting recognition that microbial diversity in the context of speciation essentially implies a defined ecological niche in terms of its life style, role in ecosystem and host preference with a shared phylogenetic heritage [37].

For a niche specific adaptation, a microbe may accentuate certain changes in its genic repertoire by undergoing substitutions in pre-existing genes, losing certain genes detrimental to a specific lifestyle, by undergoing recombination events or else by acquiring genes via lateral transfer events. In our earlier studies, we have established an important paradigm with respect to *M. avium* complex that pathogenic adaptations in *MAC*, unlike in the organisms of *M. tuberculosis* lineage, are not exclusive to the selective deletion events as shown by a congruent RD (region of deletions) profile across the pathogenic and saprophytic lineage of genus *Mycobacterium* [38]. Rather it is the selective acquisition of genes in *MAC* organisms that has helped in fine-tuning their fitness for a wide range of habitats and hosts to undergo an intracellular life style [38]. It appears that subsequent recombination events in *M. avium* lineage might have also played a key role in generating the antigenic diversity required for the differential display of pathogenicity and host range among different species. This was substantiated by our observations during analysis of DT1 (gb|L04543.1|) and DT6 (gb|L04542.1|), the genomic markers specific for *M. intracellulare* and *M. avium* lineage, respectively, for their presence and organization in *MIP* and other closely associated mycobacteria [39]. Interestingly, in *MIP*, which is the progenitor of *MAC* organisms [38], while the DT1 locus was present and organized in an uninterrupted manner, the presence of DT6 was marked by an intrusion comprised of a >2.1 kb genomic fragment (Supplementary Figure S2). However, this locus of >2.1 kb has regained a new position adjacent to DT6 in both *M. avium* subsp. *paratuberculosis* and *M. avium* subsp. *hominissuis*, albeit in opposite orientation (reverse complemented) to each other, as depicted in supplementary figure S2. It is noteworthy that this region apparently lacks any mobile element - like insertional elements or transposons thereby strongly pointing towards the role of putative recombination events in speciation in the *MAC* organisms. Thus, it can be concluded that speciation in *M. avium* complex is a direct function of genome plasticity [40] and results from a cumulative interplay of deletions, acquisitions and recombination events.

*MAC* is comprised of *M. avium* (with four subspecies namely *M. avium* subsp. *avium*, *M. avium* subsp. *paratuberculosis*, *M. avium* subsp. *silvaticum* and *M. avium* subsp. *hominissuis*), *M. intracellulare*, *M. chimaera*, *M. colombiense* and a recently emerged *M. arosiense* [12]. These organisms have a diverse host range and are mainly responsible for infections in ruminants and birds besides causing ‘opportunistic’ infections in immune compromised humans and in nosocomial settings. This group of organisms possesses extreme sequence homogeneity in their marker genes and also shares almost similar biochemical properties thereby making it exigent to differentiate them by biochemical characteristics. *M. avium* and *M. intracellulare* were universally identified and distinguished by the ability of *M. intracellulare* to cause virulence in chicken [41], thus illustrating their different immunological attributes and distinct niche preferences. Similarly, *MIP* is also defined by virtue of its unique immunological features owing to which it has been used as a commercial therapeutic vaccine against leprosy and extensive clinical trials for its efficacy against many dreaded infections and diseases like cancer [42], [43], HIV [44] and tuberculosis [7], [45] are ongoing. *MIP* reportedly does not cause any infection in mice, guinea pigs and monkeys, the animal models in which it has been tested, thus, suggesting of a saprophytic lifestyle for this bacterium [46]. Thus, *MIP* is distinctly different from the members of *MAC* including *M. intracellulare* on the basis of its unique properties as described in this study (Table 3). Moreover, analysis of a draft *MIP* genome (Saini V, Raghuvanshi S, Ahmed N et al., unpublished) indicated an average GC (G+C) content of ∼68.0% for *MIP*, which differs considerably from that of *M. avium* subsp. *paratuberculosis* (69.30%), *M. avium* subsp. *hominissuis* (69.0%), *M. avium* subsp. *avium* (69%, ACFI00000000) and that of *M. intracellulare* (67%, ABIN00000000). The genomic GC content constitutes an important paradigm in prokaryotic evolution and is critical for taxonomic analyses [47]. The deviations of more than 1% in total GC content of *MIP* both from *M. avium* lineage organisms and *M. intracellulare* reaffirms its distinctiveness from these organisms belonging to *MAC*. Hence, *MIP* should be assigned an appropriate taxonomic status as a distinct species belonging to *MAC*. Additionally, the comparative evaluation of genome size of *MIP* with that of *M. avium* subsps. *paratuberculosis* (4.82 Mb), *M. avium* subsps. *avium* (4.85 Mb), *M. avium hominissuis* (5.47 Mb) and *M. intracellulare* (5.32 Mb) revealed that *MIP* has a larger genome size (Saini V, Raghuvanshi S, Ahmed N et al., unpublished).

**Table 3:** Comparative analysis of various taxonomic attributes of *MIP vis-à-vis* other members of *MAC*.

Mycobacterial species	Phylogenetic attributes	Genomic attributes		Biochemical attributes	Nature of organism
		Size (Mb)	G+C content (%)		
*M. avium* subsp. *paratuberculosis*	Different	4.8	69.3	Different	Pathogenic
*M. avium* subsp. *hominissuis*	Different	5.4	69.0	Different	Pathogenic
*M. intracellulare*	Similar	5.3	67.0	Different	Pathogenic
*MIP*	-	>5.5	∼68.0	-	Saprophytic

*MIP* can be distinguished from all the members of *M. avium* complex owing to its different phylogenetic, biochemical, immunological and genomic features. However, in case of *M. intracellulare*, phylogenetic analysis based on marker genes does not have sufficient resolution to differentiate it from *MIP*.

These observations, in the light of the non-pathogenic nature of *MIP* coupled with our detailed genome wide studies, demonstrate that *MIP* indeed is a distinct and unique organism belonging to *MAC*. The growth pattern of *MIP* exhibited a growth rate that was faster than the typical slow growers such as *M. tuberculosis* and slower in comparison to typical fast growers, such as *M. smegmatis*, thus placing *MIP* more or less equidistant from the slow and fast growers belonging to genus *Mycobacterium*. It is noteworthy here that, in mycobacteria, fast growers normally represent non-pathogenic organisms while slow growers are usually specialized pathogens. The FAME analysis of *MIP* and its comparison with the fatty acid complement from other mycobacterial species also substantiates the placement of this saprophyte in between fast and slow growers. Thus, it appears that *MIP* represents an organism in evolutionarily transitory position with respect to a fast grower and a slow grower, a fact also reflected upon by ‘low - resolution’ of phylogenetic signals in terms of its segregation from other closely related species. Thus, *MIP* may effectively demarcate the boundaries between a philanthropic vaccine strain and seasoned pathogens like opportunists of the *MAC* lineage. The novelty of *MIP* established in this study provides a categoric evidence to formally endorse the earlier proposed name *M. indicus pranii* for this newly established mycobacterial species [9]. Hence, in future, it should be designated as *Mycobacterium indicus pranii* in the relevant databanks. The unraveling of this organism's genomic blueprint would help in understanding the evolutionary events that underpin the circuits of growth and virulence optimization in the genus *Mycobacterium*.

This study highlights that the taxonomic categorization in genus *Mycobacterium* is intricate and difficult to disentangle from rest of the taxa. The species boundaries in this genus may not be circumscribed to few changes in housekeeping genes which have a variable rate of substitution and are, often, non adequately sensitive and specific to encompass all the evolutionary events in the realms of speciation as highlighted in the present work. A uniform and consensus derived polyphasic framework based on phylogenetic, biochemical and chemotaxonomic investigations is proposed for resolving such prevalent heterogeneities in mycobacteria.

### Species Description for *M. indicus pranii* (DSM 45239^T^ = MTCC 9506^ T^)

*M. indicus pranii* (*MIP*) is a cultivable, non-pathogenic saprophytic organism, which belongs to Runyon group IV based on its growth and biochemical characteristics, summarized in table 1. It gives a smooth and round colony type (on the entire margin), size 1–2 mm and can be grown at 25°C to 45°C on Lowenstein Jensen, Dubos and MB7H11 agar, 5% NaCl and 10 µg/ml isoniazid [19]. It does not produce any pigment either in light or dark. It was found to be negative for niacin test, positive for tellurite reduction, negative for Tween 80 hydrolysis as well as negative for urease test [18], [19]. The organism did not grow either on fructose or on arabinose as the only source of carbon. It differs significantly from slow growers such as *M. tuberculosis*, *M. intracellulare*, *M. avium*, *M. chimaera* and also from fast growers like *M. fortuitum*, *M. chelonae*, *M. smegmatis* and *M. vaccae* based on a profiling of its biochemical properties. *MIP* can be typified by a summed feature III that corresponds to 20:0 alcohol/19:0 cycloprop *ω*10c and/or 19:0 cycloprop *ω*8c, comprising 67.25% of total fatty acid content analyzed, a summed feature II, which is comprised of 13.89% and consists of 17:1*ω*7c/18: 0 alcohol/17:1*ω*6c/17: cyclopropane, and a summed feature I, which represents <1% of total fatty acid analyzed and constitutes of 8-Me-16:0/10-Me-16:0. *MIP* could be differentiated from other mycobacteria by the presence of fatty acids like 18:1*ω*7c (1.37%) and absence of 20:0 fatty acids. With a GC content of ∼68%, it differs significantly from its nearest phylogenetic relatives of *MAC* and also has a considerably larger genome size as compared to *M. tuberculosis*, *M. avium* subsp. *hominissuis*, *M. avium* subsp. *paratuberculosis*, and *M. intracellulare*. The phylogenetic analysis has established *MIP* as the predecessor of *MAC* complex [38]. The type species of *MIP* is DSM 45239^T^ = MTCC 9506^T^.

## Materials and Methods

### DNA isolation and strain authentication

*MIP* was received on LJ slant as a kind gift from Dr. Rajni Rani, National Institute of Immunology, New Delhi. The culture was streaked on MB7H11 agar supplemented with 1X OADC (Oleic acid Albumin Dextrose Catalase) as well as on LB (Luria Bertani) agar to check for any contaminating bacteria. Once the purity of the culture was confirmed, it was inoculated into MB7H9 medium and the genomic DNA was isolated. Briefly, *MIP* culture (100 ml) was grown to an absorbance of 1.5 at 600 nm (A_600nm_) in MB7H9 medium supplemented with 0.5% glycerol, 0.2% Tween-80 and 1x ADC (albumin dextrose catalase) at 37°C in an orbital shaker at 200 rpm followed by incubation with glycine (1%) at 37°C for 24 hrs. After 24 hrs of the addition of glycine, cells were harvested by centrifugation at 8,000 rpm for 10 min at room temperature and were lysed by incubating first with 5 ml lysis buffer, TEG (Tris EDTA glucose) containing 500 µl lysozyme (20 mg/ml) at 37°C for 16 hrs followed by incubation with 1 ml SDS (10%) and 500 µl proteinase K (10 mg/ml; Sigma) at 55°C for 40 min with intermittent gentle swirling. The lysate was incubated with 2 ml of NaCl (5 M) and 1.6 ml of CTAB (cetyl trimethyl ammonium bromide) at 65°C for 10 min. Genomic DNA was extracted with phenol (pre-equilibrated with Tris-HCl, pH 8.0) and chloroform (1∶1) [twice] followed by chloroform extraction [twice]. DNA in the aqueous phase was precipitated by incubation with 0.6 v/v isopropanol at room temperature for 15 min. The genomic DNA spool was removed by using a sterile microtip washed with 70% ethanol, air dried and resuspended in 200 µl autoclaved double distilled water and kept at 4°C for the proper resuspension of DNA.

To ascertain the authenticity of the strain, *MIP* was tested for the presence of unique nucleotide substitutions reported for its *hsp65* gene [10]. The primers Tb11 (5′-accaacgatggtgtgtcc-3′) and Tb12 (5′-cttgtcgaaccgcatacct-3′) were used to amplify *hsp65* by PCR using *MIP* genomic DNA as template. Briefly, amplification reaction contained 50 ng of template DNA, 1x Taq polymerase buffer, 200 µM each of deoxynucleotide triphosphates (dNTP), and 1 µl of 20 pm/µl each of the primers, 1.5 mM MgCl_2_ and 2 U of Taq polymerase (NEB, UK). The amplification reaction comprised of initial denaturation at 94°C for 5 min, thirty cycles of denaturation at 94°C for 1 min, annealing at 55°C for 2 min and an extension at 72°C for 2 min followed by a final extension at 72°C for 10 min.

### Growth rate analysis

*MIP* was grown on nutrient agar, MacConkey's agar, LJ slant and OADC supplemented MB7H11 agar. *MIP* was evaluated for colony forming time on MB7H11 agar. It was also grown in MB7H9 broth supplemented with 1XADC and 0.2% Tween 80 as mentioned earlier. For all purposes, A_600nm_ was measured at appropriate time points throughout the growth of the 100 ml culture. Briefly, small aliquots of the culture (0.1 ml) were removed aseptically; diluted to 1∶10 with MB7H9 supplemented with 0.2% (v/v) Tween 80-1XADC and the A_600nm_ was measured. The A_600nm_ of *MIP* cultures were plotted against time, and a typical growth curve was generated. However, the members of *MAC* are known to grow optimally at 30°C [48]. Considering the proximity of *MIP* with *MAC*, *MIP* was also evaluated for its growth at 30°C.

### Phenotypic, Biochemical and chemotaxonomic analysis

*MIP* colonies were physically examined for their type, appearance, and morphology and pigment production. The culture features like growth at 25°C, 37°C and 45°C, pigment production, tolerance to NaCl, resistance to isoniazid were examined using standard lab procedures [49]. Whole-cell fatty acid analysis was performed by Gas Liquid Chromatography using profiles in the Microbial Identification System [26] (MIDI Inc., Newark, Del.). Mycobacterial cells were grown and harvested according to the manufacturer's protocols (MIDI Inc., Newark, Del.). Peaks were integrated automatically and fatty acid names and percentages were determined using the MIDI software package provided by manufacturers. This tool also generated distance values from the nearest organisms based on the comparison of fatty acid profiles stored in MIDI database. GC content was calculated from the whole genome data of *MIP* by using indigenously developed perl scripts.

### Molecular taxonomy, phylogenetic analysis and sequence submission

*MIP* genes encoding for 16S rRNA, *hsp65*, *rpoB*, DT1 and DT6 have been sequenced and retrieved as reported elsewhere [38]. The gene sequences corresponding to the relevant orthologues in other mycobacterial organisms were retrieved from NCBI (ftp://ftp.ncbi.nih.gov/genomes/Bacteria/). Similarity searches of current nucleotide databases were carried out with the network service of the National Center for Biotechnology Information (http://www.ncbi.nlm.nih.gov) with the BLAST algorithm and percentage mismatch in 16S rRNA, *hsp65* and *rpoB* sequences was inferred. Percentage mismatch was calculated as number of total mismatches/length of alignment into hundred. To construct phylogenetic tree, the sequences were downloaded from the gene databanks, concatenated and aligned with clustalx ver.1.81 [50]. A phylogenetic tree rooted to *M. malmoense* was constructed using Neighbour joining (NJ) method with 1,000 bootstrap iterations [51]. *MIP* sequences used in this study have been deposited to gene databanks under various accession numbers 16S rRNA (DQ437715), *hsp65* (DQ437718), *rpoB* (DQ437721) and the locus encompassing DT6 region (FJ970491).

### Culture deposition

*MIP* has been deposited at MTCC, IMTECH, Chandigarh, India (accession no. MTCC 9506^T^) and at DSMZ (German Collection of Microorganisms and Cell Cultures, Braunschweig, Germany; accession no. DSM 45239^T^).

## Supporting Information

Figure S1 Sequence Alignment of complete *rpoB* gene of *MIP*: The comparative analysis of *rpoB* of *MIP* reveals that it shares a homology of 96%, 95% and 91% with *M. avium* subsp. *hominissuis* (*MAH*), *M. avium* subsp. *paratuberculosis* (*MAP*) and *M. marinum* (*MMAR*), respectively. This suggests that *MIP* is distinct from other mycobacterial species used in this analysis [22]. The sequences were aligned with clustal x ver 1.81[50] and alignments were edited using Jalview [52]. The major regions of divergence have been boxed and are indicated by arrow marks.
(2.94 MB TIF)

Figure S2 Genomic organization of DT6 (the genomic marker specific for *M. avium* lineage): The analysis of DT6 in *MIP* and associated organisms for its presence and organization revealed that this region was marked by an intrusion comprised of a >2.1 kb genomic fragment in *MIP*, the progenitor strain of *MAC* lineage (38). However, this locus of >2.1 kb has regained a new position adjacent to DT6 in both *M. avium* subsp. *paratuberculosis* and *M. avium* subsp. *hominissuis*, albeit in opposing orientation, suggesting thereby of a putative recombination event (see the orientation and change in the sequence arrangement on the locus). The dotted lines depict the recombination within DT6 region while the straight lines show the arrangement of >2.1 kb region in these species of *MAC*.
(0.16 MB TIF)
