# Supplementary figures and images for: Polyphasic Taxonomic Analysis Establishes Mycobacterium indicus pranii as a Distinct Species
Source: PLoS One. 2009 Jul 16;4(7):e6263. doi: 10.1371/journal.pone.0006263 (PMC2707620; doi:10.1371/journal.pone.0006263)

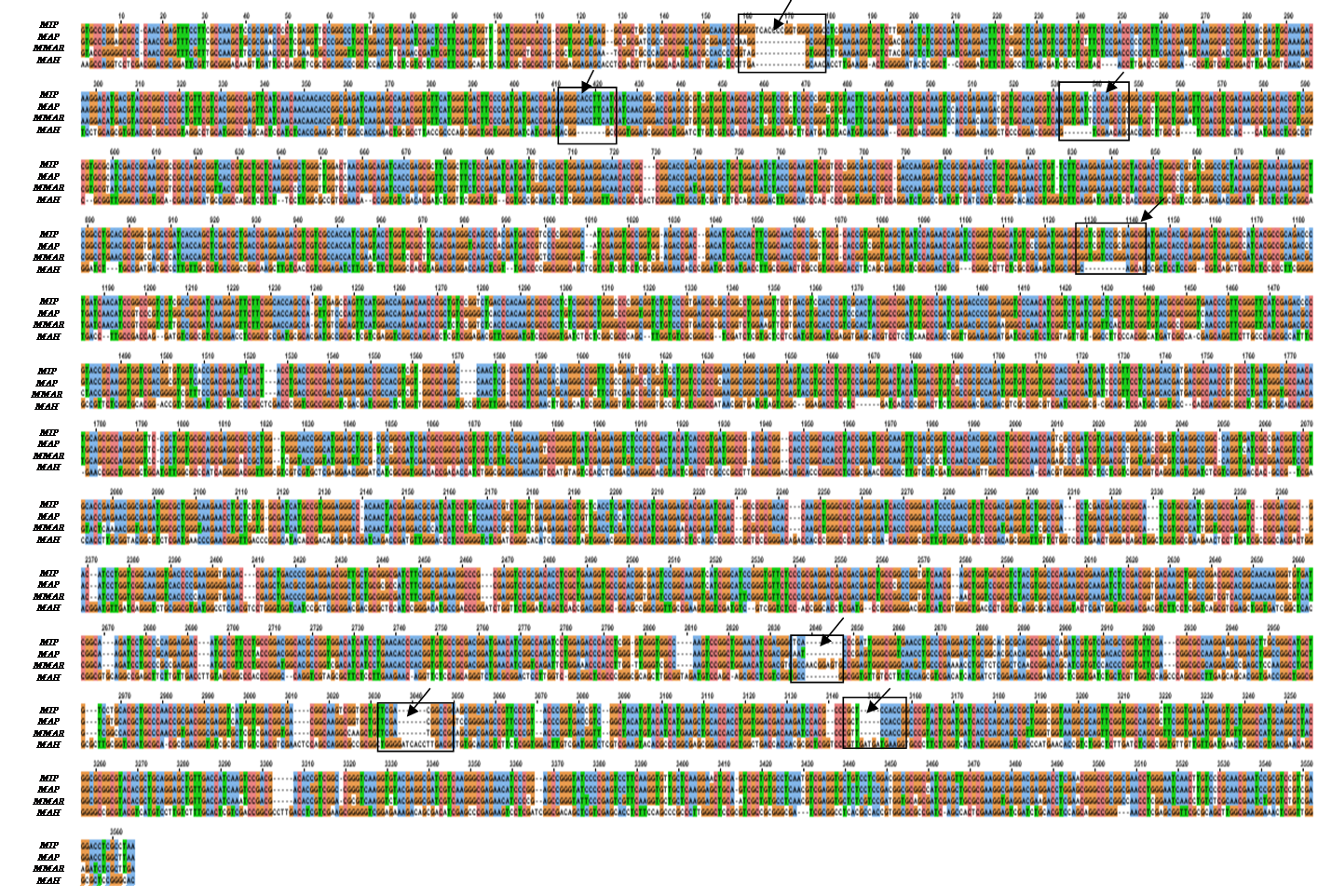

Supplement: Figure S1 — Sequence Alignment of complete rpoB gene of MIP: The comparative analysis of rpoB of MIP reveals that it shares a homology of 96%, 95% and 91% with M. avium subsp. hominissuis (MAH), M. avium subsp. paratuberculosis (MAP) and M. marinum (MMAR), respectively. This suggests that MIP is distinct from other mycobacterial species used in this analysis [22]. The sequences were aligned with clustal x ver 1.81[50] and alignments were edited using Jalview [52]. The major regions of divergence have been boxed and are indicated by arrow marks. (2.94 MB TIF) [file pone.0006263.s001.tif]

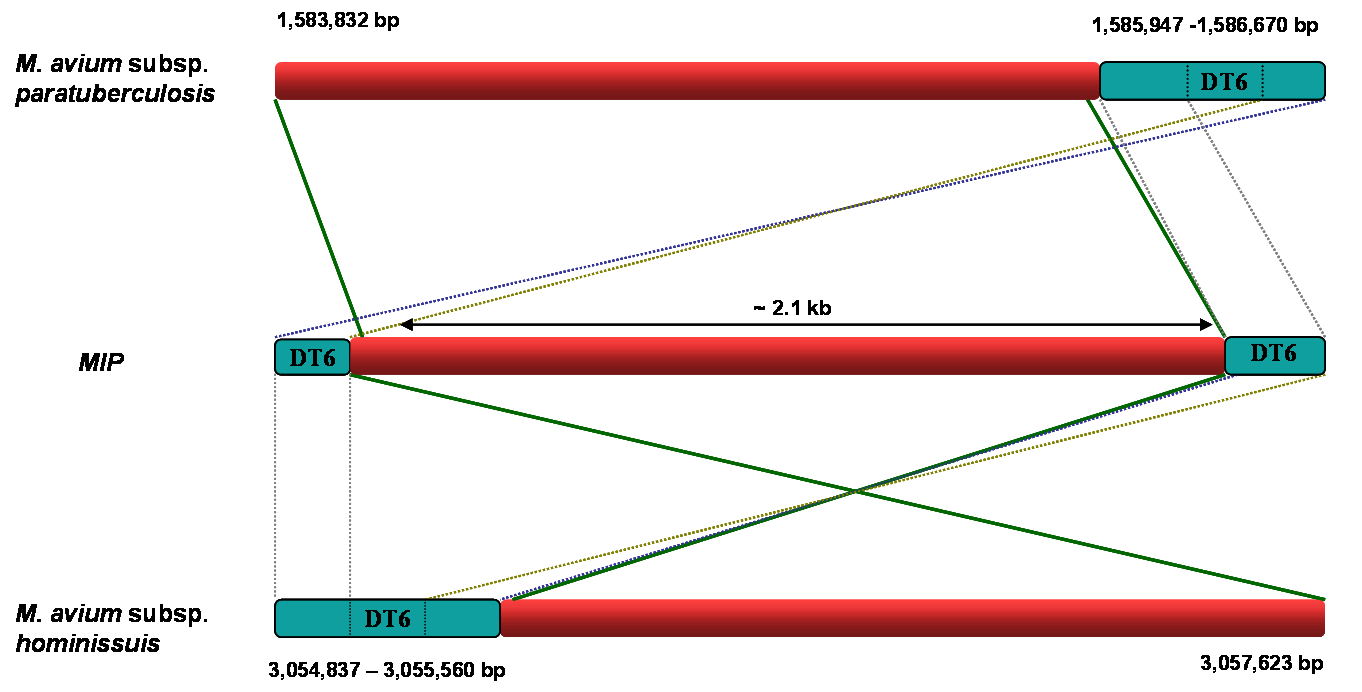

Supplement: Figure S2 — Genomic organization of DT6 (the genomic marker specific for M. avium lineage): The analysis of DT6 in MIP and associated organisms for its presence and organization revealed that this region was marked by an intrusion comprised of a >2.1 kb genomic fragment in MIP, the progenitor strain of MAC lineage (38). However, this locus of >2.1 kb has regained a new position adjacent to DT6 in both M. avium subsp. paratuberculosis and M. avium subsp. hominissuis, albeit in opposing orientation, suggesting thereby of a putative recombination event (see the orientation and change in the sequence arrangement on the locus). The dotted lines depict the recombination within DT6 region while the straight lines show the arrangement of >2.1 kb region in these species of MAC. (0.16 MB TIF) [file pone.0006263.s002.tif]
